# Data-Driven Analysis of Systemic Indicators Linking Stroke-Associated Pneumonia, Delayed Cerebral Ischemia, and Outcome After Aneurysmal Subarachnoid Hemorrhage

**DOI:** 10.3390/jcm15041359

**Published:** 2026-02-09

**Authors:** Vanessa Magdalena Swiatek, Conrad-Jakob Schiffner, Tom Tobias Kummer, Lea Ehrhardt, Klaus-Peter Stein, Ali Rashidi, Sylvia Saalfeld, Robert Werdehausen, I. Erol Sandalcioglu, Belal Neyazi

**Affiliations:** 1 Department of Neurosurgery, Otto-Von-Guericke University, 39120 Magdeburg, Germanyconrad-jakob.schiffner@med.ovgu.de (C.-J.S.); ali.rashidi@med.ovgu.de (A.R.);; 2 Department of Anesthesiology and Intensive Care Medicine, Otto-Von-Guericke University, 39120 Magdeburg, Germany; 3 Department of Data Analysis in Life Sciences, Ilmenau University of Technology, 98693 Ilmenau, Germany; lea.ehrhardt@tu-ilmenau.de; 4 Research Campus STIMULATE, 39016 Magdeburg, Germany; 5 Department of Medical Informatics, University Hospital Schleswig-Holstein Campus Kiel, 24105 Kiel, Germany

**Keywords:** aneurysmal subarachnoid hemorrhage, delayed cerebral ischemia prediction, functional outcome prediction, stroke-induced immunodepression syndrome, stroke-associated pneumonia, machine learning, longitudinal data analysis

## Abstract

**Background/Objectives**: Delayed cerebral ischemia (DCI) is a major cause of poor outcome after aneurysmal subarachnoid hemorrhage (aSAH). Beyond large-vessel vasospasm, DCI reflects a systemic, multifactorial process involving inflammation, hematologic dysregulation, and organ dysfunction. Stroke-associated pneumonia (SAP), a frequent aSAH complication linked to stroke-induced immunodepression, may aggravate secondary ischemic injury. Unlike prior studies focusing on classical predictors alone, we included pneumonia and longitudinal respiratory parameters alongside inflammatory, hematologic, and renal markers. Using machine learning, this study aimed to identify predictors of DCI and functional outcome from routinely collected intensive care data. **Methods**: In this retrospective single-center study, 182 aSAH patients treated in a neurosurgical intensive care unit were included. Clinical data, SAP status, and longitudinal inflammatory, hematologic, renal, and respiratory parameters were extracted. DCI and functional outcome were assessed. Continuous variables were summarized as minimum, maximum, and mean values. Supervised machine learning models combining 12 feature selection methods and 12 classifiers were trained using five-fold cross-validation and evaluated by accuracy, F1-score, and AUC. **Results**: DCI occurred in 22% of patients, and SAP in 27%. The machine learning models achieved a mean accuracy of 59.7% (F1-score 58.8%, AUC 59.7%) for DCI prediction. No single dominant feature emerged; predictive patterns included leukocyte counts, CRP, erythrocyte indices, platelet variability, renal function, and oxygenation metrics. Functional outcome prediction performed moderately better (mean AUC 65.7%) and shared overlapping predictors. **Conclusions**: DCI reflects systemic instability in aSAH, with longitudinal inflammatory and respiratory variability outperforming static thresholds. Dynamic risk stratification may enable earlier detection of deterioration, supporting future time-series modeling and external validation.

## 1. Introduction

The rupture of an intracranial aneurysm (IA) is the leading cause of spontaneous subarachnoid hemorrhage [1]. With an estimated global incidence of 6.7 per 100,000 persons annually, approximately 500,000 individuals are affected worldwide each year [2]. Aneurysmal subarachnoid hemorrhage (aSAH) predominantly affects younger patients, with a peak incidence between the ages of 50 and 60 [3]. Despite major advances in neurocritical care [3], 30-day mortality remains high at approximately 42%, with 10–25% of patients dying before hospital admission [4,5,6]. Among survivors, up to 50% develop long-term functional impairments, often requiring assistance in daily activities [4,7,8,9], resulting in substantial loss of productivity and economic burden on healthcare systems [4,10]. In addition to high morbidity and mortality, aSAH leads to considerable economic strain on healthcare systems [10]. The disability-adjusted life years lost due to aSAH are comparable to those caused by ischemic stroke [11,12].

The poor outcomes associated with aSAH are not solely due to the initial hemorrhagic insult but also contributed to delayed complications. Among these, delayed cerebral ischemia (DCI) is particularly feared, as it is associated with progressive neurological deterioration and worsened prognosis. Current clinical definitions consider DCI to be a new focal neurological deficit or a decline in consciousness not explained by other causes such as hydrocephalus, rebleeding, or metabolic disturbances [13]. While DCI has historically been attributed to cerebral vasospasm (CVS), it is now increasingly recognized as a multifactorial process [14,15,16]. Despite its clinical relevance, early identification of patients at risk for DCI remains a challenge.

In recent years, numerous efforts have been made to predict DCI using traditional clinical risk stratification and modern computational approaches. Established predictors include demographic patient characteristics, severity of clinical presentation, the amount of subarachnoid blood on initial CT imaging, admission laboratory markers such as C-reactive protein, hemoglobin, hypoxia-inducible factor-1α, vascular endothelial growth factor, and endothelin-1, inflammatory composite scores, and dynamic hematologic changes including early platelet decline [17,18,19,20,21,22,23,24,25]. Beyond single-parameter predictors, multivariable clinical models, nomograms, and machine learning-based approaches have been proposed, demonstrating moderate to good predictive performance [26,27,28,29,30,31,32]. Notably, most existing models rely predominantly on static or admission-level variables, thereby neglecting evolving systemic and physiological disturbances during the neurocritical care course.

More recently, longitudinal and time-resolved modeling strategies have emerged, integrating dynamic laboratory, respiratory, or physiological parameters [27,33,34]. However, these approaches primarily aim to optimize short-term predictive accuracy and rarely address the conceptual contribution of cumulative systemic instability to DCI pathogenesis. We, therefore, deliberately examined aggregated longitudinal systemic features—including inflammatory, hematologic, renal, and respiratory parameters—across the entire intensive care unit (ICU) stay, aiming to capture global physiological instability and immune–respiratory interactions that may predispose to secondary cerebral ischemia.

The increasingly complex pathophysiological understanding of DCI raises the possibility that systemic infectious and immunological disturbances may play a mechanistic role in promoting DCI. Following aneurysm rupture, blood-derived damage-associated molecules—such as free hemoglobin and heme—enter the subarachnoid space and activate innate immune signaling [35,36]. This outside-in inflammatory cascade disrupts the blood–brain barrier, promotes leukocyte infiltration, and induces the release of pro-inflammatory cytokines and reactive oxygen species, thereby exacerbating secondary brain injury [37,38,39]. Systemically, neuroendocrine and sustained sympathetic stimulation lead to lymphocyte suppression and neutrophil activation [40,41,42,43]. This immunological shift is associated with a markedly increased risk of infectious complications, most notably stroke-associated pneumonia (SAP) [44,45]. Artificial intelligence and machine learning models are increasingly used to predict and manage post-stroke infections, including SAP. These models integrate clinical and laboratory data to identify high-risk patients with good accuracy, supporting early intervention and individualized care. However, as emphasized by the American Stroke Association, most tools remain in pilot phases and require further prospective validation before routine clinical use [46,47,48,49].

Among post-aSAH infections, SAP is the most frequent and clinically impactful. It has been associated with prolonged intensive care stays, increased incidence of DCI, and worse neurological outcomes [44,50,51]. Its multifactorial pathogenesis involves impaired airway protection, reduced mucociliary clearance, and immune suppression driven by stroke-induced immunodepression syndrome (SIDS) [52]. SIDS describes the sustained suppression of systemic immune responses following acute brain injury [14,40]. Although sometimes categorized within the broader concept of central nervous system injury-induced immunodepression, SIDS more specifically reflects the immunological consequences of cerebrovascular events such as aSAH [40]. These alterations result from complex interactions between the central nervous and immune systems, mediated through neuroendocrine and autonomic pathways, which contribute to secondary complications after aSAH [53,54,55,56,57].

Given the clinical relevance of SAP as a frequent and impactful complication following aSAH, this study aimed to investigate its potential role in the development of DCI and poor functional outcome. Using a hypothesis-free machine learning approach, we analyzed longitudinally extracted clinical and physiological parameters—including inflammatory markers, hematologic indices, renal function, and oxygenation metrics—that may reflect both systemic responses to aSAH and secondary impairments related to SAP. In addition to identifying predictors of DCI and outcome, we examined the incidence of SAP within our cohort and explored its association with subsequent ischemic complications, aiming to better understand the systemic contributors to secondary brain injury.

## 2. Materials and Methods

### 2.1. Materials

This retrospective, single-center study included data from 193 neurosurgical intensive care patients with aSAH who were treated at the Department of Neurosurgery, Otto von Guericke University Magdeburg, between April 2019 and May 2024. The analysis of patient data and the applied methodology were approved by the Ethics Committee of the Medical Faculty at Otto von Guericke University (protocol number RENOVA 94/20, approval date: 22 June 2020).

#### 2.1.1. Data Collection

The analyzed parameters included demographic data (age and sex), information on cardiovascular comorbidities, malignancy or autoimmune disease, and home medication. Hemorrhage-specific variables comprised the type of cerebral hemorrhage and imaging-based characteristics such as the presence of intraventricular hemorrhage, hydrocephalus, ischemic stroke at admission, and midline shift. Aneurysm-related variables included rupture status, aneurysm location, and the presence of multiple intracranial aneurysms. Additional data encompassed the type and timing of aneurysm treatment, the occurrence and management of hydrocephalus, as well as the occurrence, diagnostic evaluation, and treatment of CVS and DCI. Clinical severity scores assessed were the Hunt and Hess grade, Fisher score, Glasgow Coma Scale, and the World Federation of Neurosurgical Societies scale at admission. Functional outcomes were evaluated using the modified Rankin Scale (mRS) at discharge.

Microbiological data were collected from respiratory and blood samples. Imaging studies included chest X-rays and, when available, chest CT scans. Further parameters comprising measured peripheral oxygen saturation (SpO_2_), complete and differential blood counts, C-reactive protein (CRP), estimated glomerular filtration rate (eGFR), and blood gas analyses with values for arterial oxygen partial pressure (paO_2_), oxygen partial pressure (pO_2_) and fraction of inspired oxygen (FiO_2_).

All parameters were measured longitudinally throughout the patients’ entire stay in the intensive care unit and manually extracted from the digital patient records. For each parameter, the minimum (lowest recorded), maximum (highest recorded), and mean (average of all recorded values) across the ICU period were calculated. SAP was diagnosed within this same observation window; however, due to the retrospective design and longitudinal aggregation, a precise temporal attribution of SAP onset relative to individual measurements was not feasible in this analysis.

#### 2.1.2. Inclusion Criteria

We included both ventilated and awake, spontaneously breathing patients with confirmed aSAH applying the following inclusion criteria:

1. Detection of subarachnoid hemorrhage caused by aneurysm rupture via CT-scan or lumbar puncture;
2. Treatment in an intensive care unit following aneurysm rupture;
3. Availability of complete clinical and imaging datasets;
4. Availability of at least two complete arterial blood gas analysis results during the acute phase of treatment;
5. Availability of at least two measurements each of the complete and/or differential blood count, C-reactive protein levels, and estimated glomerular filtration rate during the acute phase.

#### 2.1.3. Patient Cohort

After applying the five inclusion criteria mentioned above, a total of 182 patients with aSAH were included in the present study. Eleven patients were excluded due to incomplete clinical datasets. Among the included patients, 55 patients presented with multiple IA, while the remaining 127 had a single aneurysm. During hospitalization, only one patient experienced the rupture of two aneurysms, although not simultaneously. The gender distribution within the cohort was 72% female and 28% male. The mean age at the time of aneurysm rupture was 56.4 years; 79% of patients were younger than 70 years, and 21% were aged 70 years or older. An overview of the demographic data of the included patients and aneurysms can be found in Table 1.

### 2.2. Definitions

CVS was assessed clinically and through a multimodal diagnostic approach including transcranial Doppler ultrasonography, computed tomography angiography, and intra-arterial digital subtraction angiography. However, for the purposes of this study, only angiographically confirmed CVS was classified as such. Patients in whom CVS was suspected based on transcranial Doppler findings but not confirmed by angiography were classified as not having CVS. The definition of DCI was based on the criteria proposed by Vergouwen et al. [13]. Only cases in which the time of ictus could be retrospectively determined to within one week were included, based on information obtained from emergency medical service and ambulance records. All patients with aSAH received standardized prophylactic treatment to prevent DCI. This included continuous administration of nimodipine (orally or via intravenous infusion), maintenance of mean arterial pressure above 90 mmHg, normovolemic fluid management guided by laboratory parameters, and the assurance of adequate oxygenation.

The diagnosis of pneumonia in this study was based on the S3 guideline of the Association of the Scientific Medical Societies in Germany, complemented by the criteria proposed by Smith et al. (Table 2) [58].

In our cohort, all pneumonias were classified as SAP, having occurred within the first seven days following the ictus of aSAH. Due to the limitations of imaging in the intensive care setting—where chest radiographs were obtained exclusively in the supine position and in a single plane—diagnostic certainty based on radiology alone was limited. Consequently, the diagnosis of SAP was established by integrating a modified version of the clinical criteria of the S3 guideline (Table 3).

Two diagnostic categories of SAP were distinguished:

- Probable SAP: clinical criteria with microbiological confirmation in the absence of radiological confirmation of a typical infiltrate;
- Confirmed SAP: clinical criteria and radiographic evidence of pulmonary infiltration.

In all cases diagnosed as probable or confirmed SAP, alternative causes were excluded, and the supporting clinical findings were present.

For international comparability, it should be noted that our modified diagnostic framework is conceptually aligned with commonly used international definitions, including the Centers for Disease Control and Prevention and American Thoracic Society/Infectious Diseases Society of America criteria for hospital-acquired and ventilator-associated pneumonia, which similarly integrate clinical signs of infection (fever, leukocytosis), respiratory deterioration, and microbiological confirmation, with radiological findings serving as supportive but not mandatory components.

### 2.3. Methodological Framework for the Machine Learning Approach

Following data acquisition and preprocessing, relevant clinical, laboratory, and respiratory parameters were compiled and organized in Microsoft Excel (Office 2016, Microsoft Corporation, Redmond, WA, USA; https://www.microsoft.com/excel accessed on 28 April 2025). The dataset included the following traditional parameters:

1. Sex;
2. Age at diagnosis;
3. Presence of comorbidities like/vascular risk factors: hypertension, diabetes, hypercholesterolaemia, peripheral arterial disease, heart diseases, prior stroke, nicotine abuse, alcohol abuse, history of thrombosis, intake of contraception, history of malignant diseases, obesity, autoimmune disease;
4. Intake of anticoagulants or antiplatelet agents before and during treatment;
5. Findings in the initical CT-scan: type of bleeding, localization of intracerebral hemorrhage if applicable, presence of intraventricular hemorrhage, midline shift or ischemic stroke;
6. Aneurysms characteristics: Rutpure status, multipilicity, localization, shape, presence of a blep;
7. Clinical scores: Hunt and Hess score, Fisher score, Glasgow Coma Scale, and the World Federation of Neurosurgical Societies scale at admission as well as the Glasgow Coma Scale at discharge;
8. Type of aneurysm repair: Previous treatments and modality, current treatment and modality;
  - Surgical group: Type of craniotomy, presence of intraoperative rupture, application of temporary clipping, application of ICG-angiography and microdoppler, evacuation of a possible intracerebral hemorrhage, performance of decompressive hemicraniectomy, postoperative ischemic and hemorrhagic complications as well as the need for revision, occlusion of the aneurysm;
  - Endovascular group: Type of endovascular approach, number of treatments, ischemic and hemorrhagic complications as well as the need for revision, aneurysm occlusion;
9. Hydrocephalus: Presence of hydrocephalus, placement of an external ventricular drainage or a ventriculoperitoneal shunt, application of actilysis;
10. Vasospasm: Detection of CVS, implantation of an intracranial pressure or brain tissue oxygenation monitoring, need for interventional spasmolysis;
11. Follow-up data: imaging modality, aneurysm occlusion, mRS at last follow-up.

Beyond the traditional aneurysm- and aSAH-related parameters, we included the following parameters linked to SAP and infection:

1. Pneumonia-related parameters: Detection of pathogens in microbiological diagnostics from bronchoalveolar lavage or tracheal secretions, presence of SAP;
2. ICU parameters: Leukocytes, neutrophil granulocytes, immature granulocytes, eosinophil granulocytes, basophil granulocytes, sum of granulocytes, lymphocytes, monocytes, erythrocytes, hemoglobin, hematocrit, mean corpuscular volume (MCV), mean corpuscular hemoglobin (MCH), mean corpuscular hemoglobin concentration (MCHC), red cell distribution width, thrombocytes, proportion of large thrombocytes, mean thrombocytes volume, CRP, eGFR, paO_2_/FiO_2_ ratio, pO_2_, SpO_2_, FiO_2_.

To enable machine learning analysis, the original dataset was transformed: continuous variables were split into maximum, minimum, and mean values, or converted into time-to-event parameters (e.g., time to onset). In our cohort, oxygenation impairment was categorized based on the paO_2_/FiO_2_ ratio as follows: mild impairment (201–300 mmHg), moderate impairment (101–200 mmHg), and severe impairment (≤100 mmHg). A drop below these thresholds was considered clinically relevant if it persisted in consecutive analyses for at least 16 h. Variables with more than 50% missing data were excluded from further analysis. For the remaining parameters, no imputation was performed; missing values were handled by case-wise exclusion within the respective machine learning folds.

For the present analysis of the impact of the extracted factors on the occurrence of DCI, a binary classification was applied. Functional outcome was assessed using a dichotomized version of the mRS, with scores of 0–2 at discharge considered a favorable outcome and scores of 3–6 classified as unfavorable.

We applied ZScoring to the feature results in Python (version 3.11, 2025) and stored the adjusted data as an Excel table. Correlation analysis was applied to remove correlating features (Pearson correlation coefficient > 0.85) within Python [59]. Followed by a k-fold cross validation the data was split into five training and test sets. The feature reduction and classification methods were calculated for each of the five folds. Twelve feature reduction methods were applied to find the most significant features. For our analysis we decided to decrease them to ten to reduce dimensionality and avoid further correlation between features which reduced computational effort of the analysis as well. The reduction count of ten was chosen after testing multiple other factors. The feature reduction methods which were used consist of ReliefF (RF) [60], Analysis of Variance (ANOVA), Chi-squared (Chi2), Linear Regression (LIR), Logistic Regression (LOR), CART Regression (CARTR), CART Classification (CARTC), Random Forest Regression (RFR), Random Forest Classification (RFC) [61], FisherScore (Fisher), Laplacian [62] and GradientBoost (RB) [60]. Dictonaries were created for training-, test sets, feature names and positions. Different papers list these methods as suitable for the reduction in features, which is why they were used for our calculations [63,64,65]. In addition, the selection of a broad set of feature-reduction techniques was clinically motivated: systemic deterioration after aSAH can arise from heterogeneous mechanisms. These processes differ in their statistical signatures—from linear trends in inflammatory markers to non-linear fluctuations in oxygenation parameters—and may not be captured by a single method. Using complementary approaches therefore ensured that clinically relevant predictors of DCI with diverse pathophysiological backgrounds could be identified without bias toward one specific physiological domain.

For each feature reduction method, twelve classification models were trained to perform prediction calculation. The utilized classification methods include Neural Networks (NN), Decision Tree (DTREE), Support Vector Machines (SVM), K-Nearest Neighbor (KNN), Random Forest (RF), Logistic Regression (LRC), Gaussian Naive Bayes (GNB) [61], eXtreme Gradient Boosting (XGB) [60], Stochastic Gradient Descent (SGD), Bernoulli Naive Bayesian (BNB), Ensemble Bagged Trees (EBT) and AdaBoost (ABC) [61]. A simplified schematic figure illustrating the overall analytical pipeline from feature reduction to classification and model evaluation is provided in Supplementary Material S1.

The most relevant features were defined by multiple feature selection methods. These methods work with different parameters in regard to frequency threshold or ranking criterias. An explanation of the functioning of every method would go beyond the scope of this work. Detailed explanations can be found on the following websites and in the mentioned studies [60,61,62]. Despite the limited dataset size (180 samples), both the neural network and the SGD classifier using log loss achieved strong performance. The neural network benefited from its bottleneck architecture, which enabled the modeling of non-linear relationships while constraining model complexity. The SGD classifier, trained with log loss, provided a well-regularized linear model that optimized probabilistic classification and generalized well in the small-sample regime.

## 3. Results

### 3.1. Demographics

Based on the definition of CVS used in this study, 55 patients showed angiographically confirmed CVS. DCI was observed in 40 patients following aneurysm rupture (Table 4).

For the diagnosis of SAP, the presence of microbiological pathogen detection in bronchoalveolar lavage or tracheal secretions, as well as radiographic or CT evidence of pulmonary infiltrates, was considered. In 50 patients, pneumonia-causing pathogens were identified in bronchoalveolar lavage or tracheal secretions. A total of 55 patients showed positive imaging findings. Accordingly, the diagnosis of probable SAP was made in 17 patients (9%), and confirmed SAP in 33 patients (18%) (Table 5).

### 3.2. Results of the Machine Learning Analysis

#### 3.2.1. Occurrence of DCI

For the prediction of the DCI classification methods like NN, DTREE and SGD and feature selection methods like LOR and RB more often presented the highest results (Supplementary Material S2). To assess the occurrence of DCI, a binary classification model was applied. Across two independent runs, the models achieved accuracies of 58.9% and 60.6%, respectively. Corresponding F1-scores were 57.5% and 60.0%, and the area under the receiver operating characteristic curve (ROC AUC) was 58.9% and 60.6%. These results indicate limited but consistent predictive performance for distinguishing between patients with and without DCI based on the selected features. While an AUC of approximately 0.60 reflects only modest discrimination at the individual-patient level, it is noteworthy that performance remained stable across independent runs despite substantial clinical heterogeneity and the absence of explicit temporal modeling. The observed performance suggests that systemic and respiratory instability carries a reproducible, though weak, signal for DCI risk, rather than serving as a standalone diagnostic classifier (Table 6).

**Figure 1:**
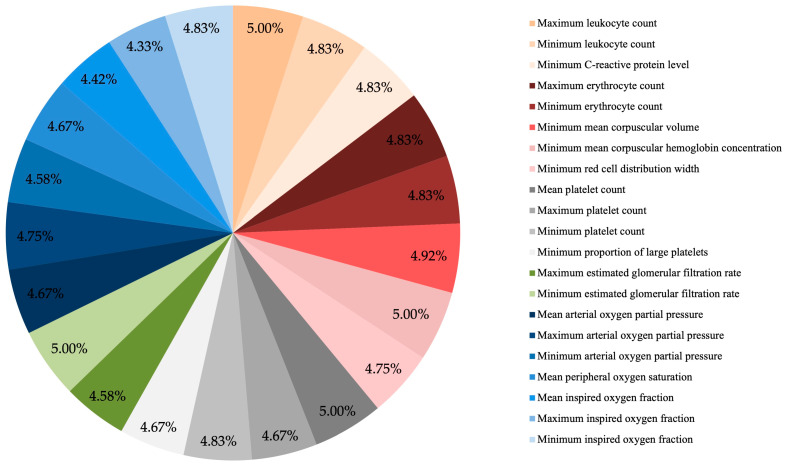
Distribution of the 21 most frequently selected features contributing to the prediction of DCI. Each segment represents the relative frequency with which a variable was selected across all feature selection methods and cross-validation runs. Features are color-coded by physiological domain: inflammatory markers (orange); red blood cell indices (red); platelet-related parameters (grey); renal function (green) and oxygenation parameters (blue). The balanced contribution across domains and the absence of a single dominant feature highlight that DCI risk reflects global systemic instability rather than reliance on any isolated parameter. Due to rounding, percentages may not total 100%.

**Figure 2:**
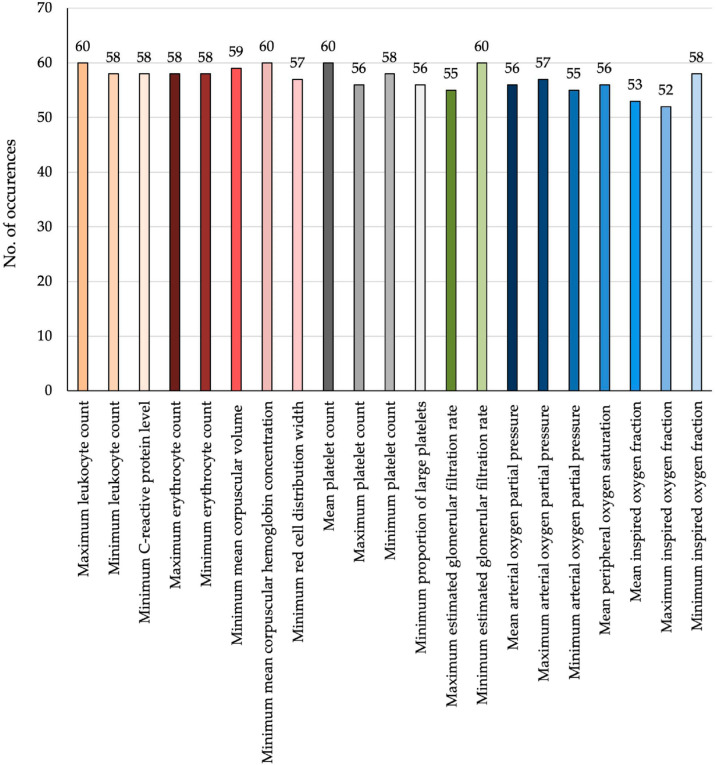
Frequency of feature selection across all models used for the prediction of DCI. Bars represent the number of times each variable was identified as relevant by the applied feature selection methods across cross-validation runs. Features are color-coded by physiological domain, including inflammatory markers (orange), red blood cell indices (red), platelet-related parameters (grey), renal function (green), and oxygenation metrics (blue). The consistently high selection frequencies across multiple domains underscore that DCI development is associated with global systemic instability rather than driven by a single physiological process.

Among the most relevant features, no single parameter emerged as dominant. Instead, a broad range of variables (*n* = 21) from different physiological domains contributed to the prediction, as outlined below (Figure 1 and Figure 2):

- Inflammatory markers (orange): maximum and minimum leukocyte counts; minimum CRP;
- Red blood cell indices (red): maximum and minimum erythrocyte counts; MCV; MCHC; minimum red cell distribution width;
- Platelet-related parameters (grey): maximum, mean, and minimum platelet counts; minimum proportion of large platelets;
- Renal function (green): minimum and maximum eGFR;
- Oxygenation parameters (blue): minimum, mean, and maximum values of pO_2_; mean SpO_2_; minimum, mean, and maximum FiO_2_.

#### 3.2.2. Functional Outcome

**Figure 3:**
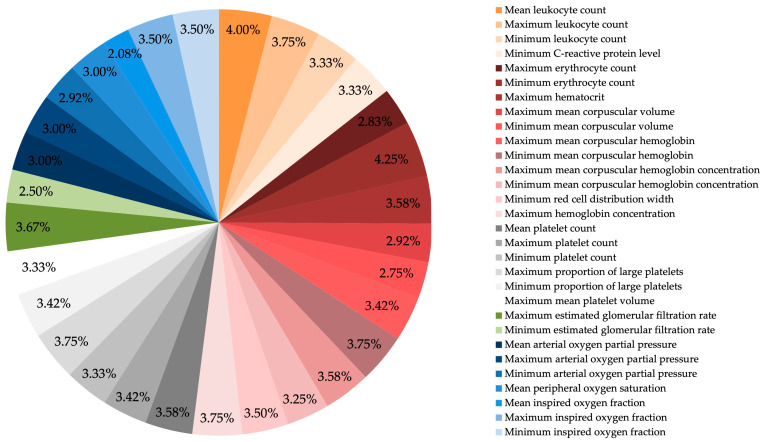
Proportional distribution of the 27 most frequently selected features contributing to functional outcome prediction after aSAH. Each segment represents the relative frequency with which a variable was selected across all feature selection methods and cross-validation runs. Features are color-coded by physiological domain: inflammatory markers (orange); red blood cell indices (red); platelet-related parameters (grey); renal function (green) and oxygenation parameters (blue). The broad and balanced contribution across physiological domains indicates that poor functional outcome after aSAH reflects cumulative systemic stress rather than impairment of a single organ system. Due to rounding, percentages may not total 100 %.

**Figure 4:**
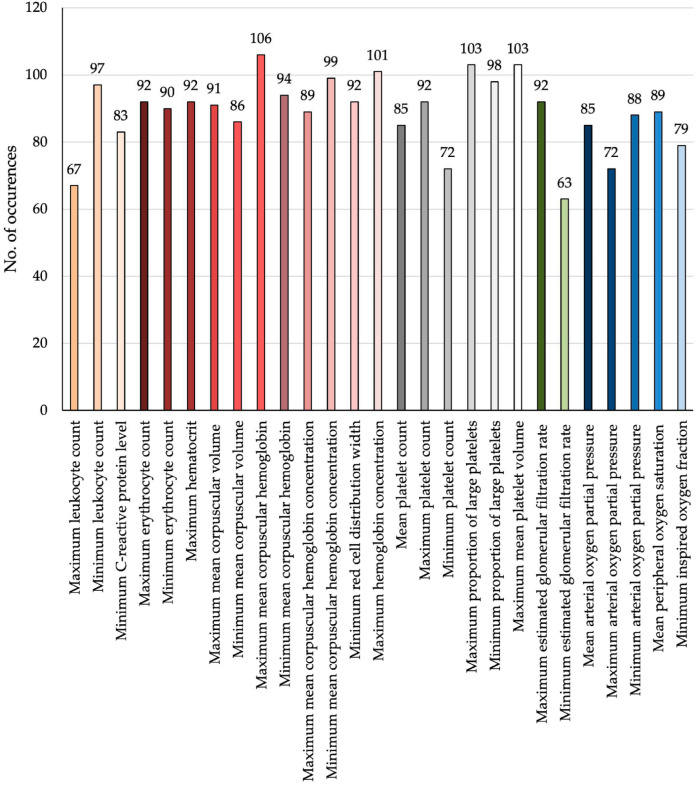
Frequency of feature selection across all models used for the prediction of functional outcome at discharge after aSAH. Bars represent the number of times each variable was identified as relevant by the applied feature selection methods across cross-validation runs. Features are color-coded by physiological domain: inflammatory markers (orange); red blood cell indices (red); platelet-related parameters (grey); renal function (green) and oxygenation parameters (blue). The broad distribution of frequently selected features across domains indicates that functional outcome after aSAH reflects cumulative systemic stress rather than impairment of a single physiological system.

To predict dichotomized functional outcomes at discharge based on the mRS, a supervised machine learning approach was implemented. For the prediction of mRS classification methods like NN and KNN and feature selection methods like RelieF, ANOVA and RFR resulted in the highest metric results in general (Supplementary Material S2). Feature selection was performed using a combination of ANOVA, RFR, and recursive feature elimination with RF, resulting in a final set of 27 variables. The most predictive features comprised a broad range of physiological parameters, overlapping significantly with those identified in the DCI analysis (Figure 3 and Figure 4):

- Inflammatory markers (orange): minimum, and maximum leukocyte counts; minimum CRP;
- Red blood cell indices (red): maximum and minimum erythrocyte counts, maximum hematocrit, maximum and minimum MCV, maximum and minimum MCH, maximum and minimum MCHC, minimum red cell distribution width, and mean hemoglobin concentration;
- Platelet-related parameters (grey): maximum, minimum, and mean platelet counts; maximum and minimum proportion of large platelets; maximum mean platelet volume;
- Renal function (green): maximum and minimum eGFR;
- Oxygenation parameters (blue): maximum, minimum, and mean pO_2_; mean SpO_2_; and minimum FiO_2_.

For the prediction of functional outcome at discharge, the best-performing models achieved a mean accuracy of 66.3%, a mean F1-score of 65.5%, and a mean area under the ROC curve of 66.1% across two independent runs. This corresponds to a moderate ability to distinguish patients with favorable functional recovery (mRS 0–2) from those with unfavorable outcomes (mRS 3–6). While not sufficient for individual outcome prediction, the results indicate that routinely collected systemic and respiratory parameters capture clinically relevant information related to early functional recovery after aSAH (Table 7).

## 4. Discussion

In this retrospective single-center study, we analyzed longitudinal clinical, laboratory, and respiratory data from 182 patients with aSAH to identify predictors of DCI and functional outcome at discharge. The dataset included inflammatory markers, oxygenation parameters, hematologic indices, renal function, and microbiological findings. Using a hypothesis-free machine learning approach, we aimed to explore whether these parameters—reflecting systemic and respiratory status—could serve as predictors of secondary complications in the context of SIDS and SAP. Within our cohort, DCI occurred in 22% of patients, which aligns with previously reported rates ranging between 20% and 30% [66]. SAP—classified as probable or confirmed—was diagnosed in 27%, affecting more than one in four individuals. These findings confirm the high prevalence of both DCI and SAP following aSAH and underscore the relevance of systemic infectious and immunological factors in shaping neurological outcomes. However, given the retrospective design and the aggregation of longitudinal parameters, these associations should be interpreted as reflecting statistical relationships and systemic risk signatures rather than causal mechanisms. Accordingly, the identified features should be regarded as risk markers of physiological instability rather than as modifiable causal risk factors.

To explore predictors of DCI, we applied a supervised machine learning approach using binary classification models based on longitudinal clinical, laboratory, and respiratory data. While the predictive performance for DCI was moderate (mean accuracy, F1-score, and AUC ~60%), this was an anticipated consequence of our conceptual study design, which deliberately prioritized the identification of cumulative systemic instability over short-term individual risk stratification. The models captured consistent patterns across independent runs and were deliberately designed to reflect systemic instability rather than isolated cerebral mechanisms. Notably, our model incorporated a broad range of systemic variables—including inflammatory markers, hematologic indices, renal function, and oxygenation parameters—and relied on repeated, time-resolved measurements, thus reflecting the dynamic nature of ICU care.

Importantly, our approach was not designed to compete with existing high-performance prediction models, but rather to provide complementary pathophysiological insight into the systemic contributors of DCI. A recent meta-analysis by Zhang et al. summarized 71 machine learning models across 48 studies and reported strong overall performance for DCI prediction, with a mean concordance index of 0.786 in training cohorts and 0.767 in validation datasets [29]. Notably, most of the included models relied predominantly on static or admission-level variables, such as clinical severity scores, radiographic findings, or baseline laboratory values, which limits their ability to capture evolving systemic and physiological disturbances during the neurocritical care course.

Willms et al. extended this paradigm by integrating static admission data with high-resolution, time-resolved laboratory and respiratory parameters collected during the first 14 days after ictus [27]. Their ensemble model, integrating logistic regression, random forest, and gradient boosting classifiers, achieved a mean AUC of 0.73 for the combined static-dynamic model and 0.66 ± 0.08 for the dynamic-only component. Notably, temporal trends in blood gas parameters (e.g., pO_2_, pCO_2_) and serum electrolytes emerged as the most influential predictors of DCI [27].

Our approach differs from both strategies in its conceptual focus. Rather than optimizing short-term predictive accuracy through static risk factors or high-frequency temporal modeling, we deliberately examined aggregated longitudinal systemic features, including inflammatory markers, hematologic indices, renal function, and oxygenation parameters, summarized across the entire ICU stay. Although this resulted in lower discrimination (AUC ~0.60), it enabled identification of cumulative systemic instability and immune–respiratory interactions that are not captured by admission-based models or short-term trend analyses alone. In this regard, our findings conceptualize DCI as a manifestation of global physiological instability rather than an isolated cerebrovascular event, and complement existing prediction models by highlighting extracerebral and systemic contributors to DCI vulnerability, rather than serving as a competing high-performance risk score. Importantly, the use of aggregated minimum, maximum, and mean values inherently limits temporal resolution and precludes causal inference. Consequently, the identified associations should not be interpreted as evidence of direct mechanistic contribution, but rather as indicators of systemic vulnerability and evolving physiological stress.

A closer examination of the most frequently selected features highlights the relevance of inflammatory markers, particularly the maximum and minimum leukocyte counts, in predicting DCI. This suggests that both excessive and suppressed immune responses may confer risk—supporting the concept of dysregulated immunity in SIDS. This aligns with findings by Zhang et al., who showed that a composite inflammatory score incorporating leukocyte subtypes, lactate dehydrogenase, and red blood cell distribution width was independently associated with DCI and mediated its effect on poor outcome [24]. Interestingly, minimum CRP emerged as a predictive feature in our model. While elevated CRP has been linked to increased risk in prior studies—such as the work by Li et al., who found CRP to be an independent predictor of vasospasm (AUC 0.81) and DCI (AUC 0.69) [22]—our findings suggest that persistently low CRP may reflect immune suppression and also indicate vulnerability to DCI. These results underscore that both hyper- and hypo-inflammatory states may be deleterious after aSAH, and that temporal immune variability—rather than absolute values—could be a key factor in risk stratification.

Similarly, red blood cell indices—particularly the maximum and minimum erythrocyte counts—were frequently selected in our model, suggesting that both anemia and erythrocytosis may modulate the risk of DCI. Additional features, including low MCV and low MCHC, indicated microcytic, hypochromic erythrocytes, while a narrow red cell distribution width pointed to a uniform, small erythrocyte population. This profile is consistent with anemia, which appears associated with increased DCI risk in our cohort. These findings are strongly supported by the study of Atakan et al., who found that patients with hemoglobin levels < 10.75 g/dL had a significantly higher incidence of DCI, with a threshold hemoglobin level showing 66% sensitivity and 66.2% specificity for DCI prediction (area under the curve: 0.68) [21]. Their results echo a broader body of evidence demonstrating that low hemoglobin levels impair cerebral oxygen delivery, increase nitric oxide scavenging through free oxyhemoglobin, and thus promote vasoconstriction and ischemia [67]. Notably, several studies have shown that maintaining Hb above 10–11 g/dL is associated with reduced infarction and lower DCI incidence [67,68,69,70,71]. Together, these findings suggest that not only absolute anemia but also subtle variations in erythrocyte morphology and volume may contribute to DCI pathogenesis through impaired oxygen transport and microvascular dysfunction.

Platelet-related parameters were among the most frequently selected features in our model, with maximum, minimum, and mean platelet counts, as well as platelet size distribution, all contributing to DCI prediction. This highlights the potential relevance of thrombocyte variability rather than absolute counts alone. Interestingly, a low proportion of large platelets, usually considered physiologically normal, was also associated with increased DCI risk—possibly reflecting impaired platelet regeneration or blunted activation responses. Our findings align with the results of Oros et al., who demonstrated that a >12.6% decrease in thrombocyte count within the first seven days after aSAH was independently associated with a 10.6-fold increased risk of cerebral vasospasm and a 2.7-fold increased risk of DCI, with the extent of decrease correlating with earlier onset of vasospasm [25]. Their study supports the hypothesis that platelet depletion reflects an early systemic response, possibly linked to inflammatory activation, thromboxane-mediated vasoconstriction, and microthrombosis, all of which are implicated in the pathogenesis of DCI [25,72,73,74]. Taken together, both our results and previous evidence suggest that early dynamic changes in platelet profiles may serve as accessible biomarkers for secondary cerebral complications after aSAH and could help identify patients at risk during the diagnostic window before DCI manifests clinically.

Oxygenation-related parameters—including minimum, mean, and maximum values of paO_2_, FiO_2_, and SpO_2_—were consistently selected as predictive features of DCI in our model. This suggests that variability in oxygenation, rather than isolated low or high values, may be indicative of systemic instability relevant to cerebral ischemic risk. This variability likely reflects the clinical heterogeneity in our cohort, which included both mechanically ventilated and spontaneously breathing patients. In this context, SAP may represent a clinically relevant risk marker of systemic inflammation, immune dysregulation, and respiratory compromise, potentially reflecting biological processes that amplify cerebral vulnerability to ischemic injury. However, given the aggregated nature of our data, this association should not be interpreted as proof of causality, but rather as an indicator of converging systemic stress pathways. Importantly, the requirement for microbiological confirmation may have preferentially identified more severe SAP phenotypes, while under-detecting clinically relevant but culture-negative pneumonia. This selection bias could have shifted the observed associations toward more pronounced respiratory dysfunction and oxygenation instability, thereby potentially amplifying the strength of the relationship between SAP-related features and DCI. Accordingly, our findings should be interpreted as reflecting the impact of clinically significant pulmonary complications rather than the full spectrum of post-aSAH infectious morbidity.

In ventilated individuals, fluctuations in paO_2_ and SpO_2_ despite controlled FiO_2_ may reflect episodes of oxygenation failure, ventilator-induced lung injury, or systemic inflammation, while in non-ventilated patients, they may indicate subclinical hypoventilation, impaired respiratory reserve, or evolving pulmonary complications. Although neither Su et al. nor Chen et al. investigated longitudinal respiratory indices, their findings support the clinical significance of respiratory and inflammatory dysfunction in the context of aSAH. Su et al. showed that in patients with aneurysmal subarachnoid hemorrhage complicated by acute lung injury, high positive end-expiratory pressure and elevated inflammatory markers were independently associated with an increased risk of DCI, possibly through impaired cerebral perfusion and endothelial dysfunction [75]. Similarly, Chen et al. identified elevated lactate dehydrogenase, neutrophil counts, and systemic inflammatory indices as strong predictors of early postoperative pneumonia, which was itself significantly associated with DCI [76]. Taken together, these results highlight the close interplay between pulmonary and cerebral physiology in aSAH. Fluctuations in oxygenation parameters in our model may therefore act as surrogate markers for underlying respiratory pathology, systemic inflammation, or ventilator-induced hemodynamic shifts that contribute to DCI.

Taken together, this convergent feature pattern supports the concept of DCI as a manifestation of global physiological instability, integrating immune dysregulation, impaired oxygen transport, microcirculatory dysfunction, and organ stress, rather than an isolated cerebrovascular phenomenon. Accordingly, the identified parameters should be interpreted primarily as biomarkers of risk and systemic instability, rather than as direct therapeutic targets, underscoring the need for prospective, time-resolved studies to disentangle temporal relationships and causal mechanisms.

While the prediction models for DCI and functional outcome at discharge (based on the mRS) shared a largely overlapping set of features, several important differences emerged. Overall, the mRS model demonstrated slightly higher predictive performance (mean AUC 65.7%) compared to the DCI model (mean AUC 59.7%), suggesting that the included parameters were more reflective of the patient’s overall recovery trajectory than of the more specific complication of DCI. Feature selection revealed that the mRS model incorporated 27 variables, compared to 21 in the DCI model. Among the features exclusively selected for mRS prediction were several additional red blood cell indices—including maximum hematocrit, maximum MCV, maximum and minimum MCH, maximum MCHC, and maximum hemoglobin concentration—as well as platelet-related markers such as maximum mean platelet volume and both maximum and mean proportions of large platelets. These features likely reflect broader aspects of systemic physiological stress, anemia, and hematologic compensation that influence functional recovery. In contrast, maximum and mean values of the FiO_2_ were selected only in the DCI model, possibly indicating that acute respiratory demands and oxygenation dynamics are more tightly linked to secondary ischemic complications than to long-term neurological outcome. Together, these findings support the notion that while DCI and poor outcome after aSAH share pathophysiological pathways, functional outcome is shaped by a more complex and cumulative systemic profile.

From a translational perspective, our findings suggest that longitudinal patterns of systemic instability—rather than isolated threshold violations—may provide clinically meaningful signals for identifying patients at increased risk of secondary deterioration. In the ICU setting, this concept could support trend-based vigilance strategies, in which patients exhibiting recurrent or oscillating abnormalities in inflammatory markers, hematologic indices, renal function, and oxygenation parameters are flagged for closer clinical surveillance and intensified multimodal monitoring, rather than triggering immediate therapeutic interventions.

Moreover, the identified feature patterns provide a data-driven foundation for future prospective studies and the development of automated alert systems based on continuous trend analysis and dynamic risk profiling. Such systems could integrate routinely collected ICU data to detect evolving physiological instability at an early stage, thereby enabling proactive diagnostic evaluation and individualized monitoring strategies. Importantly, these concepts are intended to inform future research and clinical decision support tools, rather than to suggest immediate changes to established clinical practice.

This study has several limitations that should be considered when interpreting the results. First, it was conducted as a retrospective single-center analysis, which may limit the generalizability of the findings to other patient populations or institutional practices. Functional outcome was assessed using the mRS at discharge. While this represents a pragmatic and clinically relevant early outcome measure, it may underestimate late neurological recovery, particularly in younger aSAH patients with prolonged rehabilitation potential. We selected discharge mRS as the primary outcome measure due to incomplete long-term follow-up data and loss to follow-up in a subset of patients, which precluded reliable longitudinal outcome assessment. Future prospective studies with standardized long-term follow-up are needed to better capture delayed functional improvement and late recovery trajectories. Second, although we applied a robust machine learning framework using multiple classifiers and feature selection methods, the overall predictive performance for DCI remained modest, with area under the curve values around 60%. This reflects the complexity of the underlying pathophysiology and the likely need for additional, possibly non-linear or time-resolved inputs to improve prediction accuracy. Third, the definition of pneumonia relied heavily on microbiological confirmation, which may have resulted in underdiagnosis of clinically relevant but culture-negative cases. Conversely, given the challenges of imaging in the ICU (e.g., single-plane supine chest radiographs), radiological confirmation of infiltrates was often not possible, which may have impacted classification accuracy of SAP. Fourth, although we used longitudinal data (e.g., max, min, and mean values), our analysis did not capture true time-series trajectories or temporal interdependencies between variables, such as timing of SAP onset relative to DCI. This temporal information may carry additional prognostic value and should be considered in future modeling efforts. Fifth, external validation was not performed, and the relatively small cohort size may have limited the stability of some models. A larger multicenter dataset with external validation would help confirm the robustness and clinical applicability of the findings. Finally, our cohort included both ventilated and non-ventilated patients, which introduces heterogeneity in oxygenation parameters. Although this reflects real-world ICU populations, stratified analyses might yield more precise insights into the interplay between respiratory support, SAP, and cerebral ischemia.

## 5. Conclusions

This study demonstrates that DCI and poor functional outcome after aSAH are associated with a broad range of systemic and respiratory factors. By applying a machine learning approach to longitudinal intensive care data, we identified predictive patterns involving inflammatory markers, hematologic indices, renal function, and oxygenation parameters. Importantly, our findings indicate that fluctuations and recurrent deviations in these parameters over time are more informative than isolated threshold violations, emphasizing the clinical relevance of physiological instability in the development of secondary complications. Given the aggregated nature of the data, these associations should be interpreted as non-causal and not temporally directional.

From a practical perspective, this suggests that repeated or oscillating changes in routinely measured variables, rather than single abnormal values, should prompt increased clinical vigilance. In particular, dynamic changes in leukocyte counts and CRP, variability in hemoglobin and platelet-related parameters, shifts in renal function, and unstable oxygenation requirements (paO_2_, FiO_2_, SpO_2_) emerged as relevant signals and may warrant closer monitoring in patients at risk for DCI. The frequent selection of features related to SAP and immune suppression further supports the importance of early detection and proactive management of extracerebral complications, while not implying a direct causal contribution to DCI.

Although the predictive performance of our model was moderate, the findings highlight the potential of longitudinal, multidimensional data to support trend-based risk assessment in the neurocritical care setting. Future studies incorporating true time-series modeling, automated detection of instability patterns, and external validation are needed to translate these concepts into clinically actionable monitoring tools and more personalized treatment strategies for patients with aSAH.

## Figures and Tables

**Table 1:** Patient-related and aneurysm-related epidemiological characteristics of the cohort.

Patient-Related Epidemiological Characteristics			
		*N*	%
Sex	Male	51	28
	Female	131	72
Age	<70 years	144	79
	≥70 years	38	21
Mean age (years)		56.4	
Vascular risk factors	Hypertension	111	61
	Diabetes type 2	12	7
	Hyperlipidaemia	36	20
	Nicotine abuse	72	40
	Alcohol abuse	19	10
	Obesity	29	16
	History of vascular diseases *	27	15
Hunt and Hess score	1	7	4
	2	79	44
	3	36	20
	4	17	9
	5	41	23
Aneurysm-related epidemiological characteristics			
		*N*	%
Number of aneurysms	Single	127	70
	Multiple	55	30
Localization	Anterior cerebral artery	2	1
	Anterior communicating artery	68	37
	Pericallosal artery	5	3
	Middel cerebral artery	35	19
	Internal carotid artery	29	16
	Posterior communicating artery	12	7
	Basilar artery	15	8
	Posterior inferior cerebellar artery	10	6
	Others	6	3
Aneurysm treatment	Endovascular treatment	108	59
	Surgical treatment	59	33
	No treatment	15	8

* Including peripheral arterial diseases, coronary heart disease or myocardial infarction and stroke.

**Table 2:** Overview of the diagnostic criteria for pneumonia according to Smith et al. [58]; the bold criterion is mandatory, and at least two of the remaining (non-bold) criteria must additionally be fulfilled.

Criteria	Description
**Infiltrate**	**presence of a new, persistent, or progressive infiltrate on chest radiography or computed tomography**
Leukocytes	leukocyte count < 4000/µL or >10,000/µL
Fever	body temperature > 38.3 °C
Bronchial secretion	purulent bronchial secretions
Microbiological conformation	microbiological confirmation based on pathogen detection in lower respiratory tract samples obtained via bronchoalveolar lavage or tracheal secretions

**Table 3:** Modified diagnostic criteria used for the identification of SAP in the present cohort. Microbiological confirmation was mandatory due to limited radiological diagnostic certainty in the intensive care setting. The bold criterion is mandatory for definition of SAP.

Criteria	Description
**Microbiological confirmation**	**microbiological confirmation based on pathogen detection in lower respiratory tract samples obtained via bronchoalveolar lavage or tracheal secretions**
Leukocytes	leukocyte count < 4000/µL or >10,000/µL
Fever	body temperature > 38.3 °C
Bronchial secretion	purulent bronchial secretions
Respiratory deterioration	new or worsening respiratory symptoms increased oxygen requirements decrease in the paO_2_/FiO_2_ ratio below 240

**Table 4:** Incidence of angiographically confirmed CVS and DCI among patients with aSAH.

	Yes (*N*/%)	No (*N*/%)
Angiographically confirmed CVS	55/30	122/70
DCI	40/22	142/78

**Table 5:** Microbiological and radiological findings used for the diagnosis of SAP. The table shows the number and percentage of patients with pathogen detection in bronchoalveolar lavage or tracheal secretions, radiological evidence of pulmonary infiltrates, and the resulting classification into probable or confirmed SAP.

		Yes (*N*/%)	No *(N*/%)
Detection of germs in bronchioalveolar lavage or tracheal secretions		50/28	132/72
		*N*/%	
Chest X-ray/CT findings	Positive	55/30	
	Negative	113/62	
	No radiological imaging	14/8	
Diagnosis of SAP	Probable SAP	17/9	
	Confirmed SAP	33/18	

**Table 6:** Performance metrics of the binary classification model for predicting delayed cerebral ischemia. Accuracy, F1-score, and area under the ROC curve are reported for two independent runs and as mean values. The model demonstrates limited but reproducible discrimination, consistent with the multifactorial and dynamic nature of DCI and the use of aggregated longitudinal systemic parameters.

	Accuracy	F1-Score	ROC AUC
1. Run	0.5889	0.575	0.5889
2. Run	0.6056	0.6	0.6056
Mean	0.59725	0.5875	0.59725

**Table 7:** Predictive performance for functional outcome at discharge after aSAH. Accuracy, F1-score, and area under the ROC curve are shown for two independent runs and as mean values. The model demonstrates moderate discrimination between favorable (mRS 0–2) and unfavorable (mRS 3–6) functional outcomes using longitudinal clinical and physiological data.

	Accuracy	F1-Score	ROC AUC
1. Run	0.6685	0.665	0.669
2. Run	0.6519	0.645	0.6527
Mean	0.6632	0.655	0.6609

## Data Availability

The dataset supporting the conclusions of this article is included within the article.

## Supplementary Materials

The following supporting information can be downloaded at: https://www.mdpi.com/article/10.3390/jcm15041359/s1, The Supplementary Materials include S1: a schematic illustration of the analytical pipeline from feature reduction to classification and model evaluation, and S2: a comparative overview of classifier performance and feature selection for the prediction of DCI and functional outcome (mRS), illustrating that overall predictive performance was largely classifier-agnostic, with slight method-dependent differences across models.

## Abbreviations

The following abbreviations are used in this manuscript:

IA	Intracranial aneurysm
aSAH	Aneurysmal subarachnoid hemorrhage
DCI	Delayed cerebral ischemia
CVS	Cerebral vasospasm
SAP	Stroke-associated pneumonia
SIDS	Stroke-induced immunodepression syndrome
mRS	Modified Rankin scale
SpO_2_	Peripheral oxygen saturation
CRP	C-reactive protein
eGFR	Estimated glomerular filtration rate
paO_2_	Arterial oxygen partial pressure
pO_2_	Partial pressure of oxygen
FiO_2_	Fraction of inspired oxygen
ICU	Intensive care unit
MCV	Mean corpuscular volume
MCH	Mean corpuscular hemoglobin
MCHC	Mean corpuscular hemoglobin concentration
AUC	Area under the curve
ROC AUC	Area under the receiver operating characteristic curve
ANOVA	Analysis of Variance
Chi2	Chi-squared
LIR	Linear Regression
LOR	Logistic Regression
CARTR	CART Regression
CARTC	CART Classification
RFR	Random Forest Regression
RFC	Random Forest Classification
Fisher	FisherScore
RB	GradientBoost
NN	Neural Networks
DTREE	Decision Tree
SVM	Support Vector Machines
KNN	K-Nearest Neighbor
RF	Random Forest
LRC	Logistic Regression Modell
GNB	Gaussian Naive Bayes
XGB	eXtreme Gradient Boosting
SGD	Stochastic Gradient Descent
BNB	Bernoulli Naive Bayesian
EBT	Ensemble Bagged Trees
ABC	AdaBoost
