# Supplementary figures and images for: Data-Driven Analysis of Systemic Indicators Linking Stroke-Associated Pneumonia, Delayed Cerebral Ischemia, and Outcome After Aneurysmal Subarachnoid Hemorrhage
Source: J Clin Med. 2026 Feb 9;15(4):1359. doi: 10.3390/jcm15041359 (PMC12942558; doi:10.3390/jcm15041359)

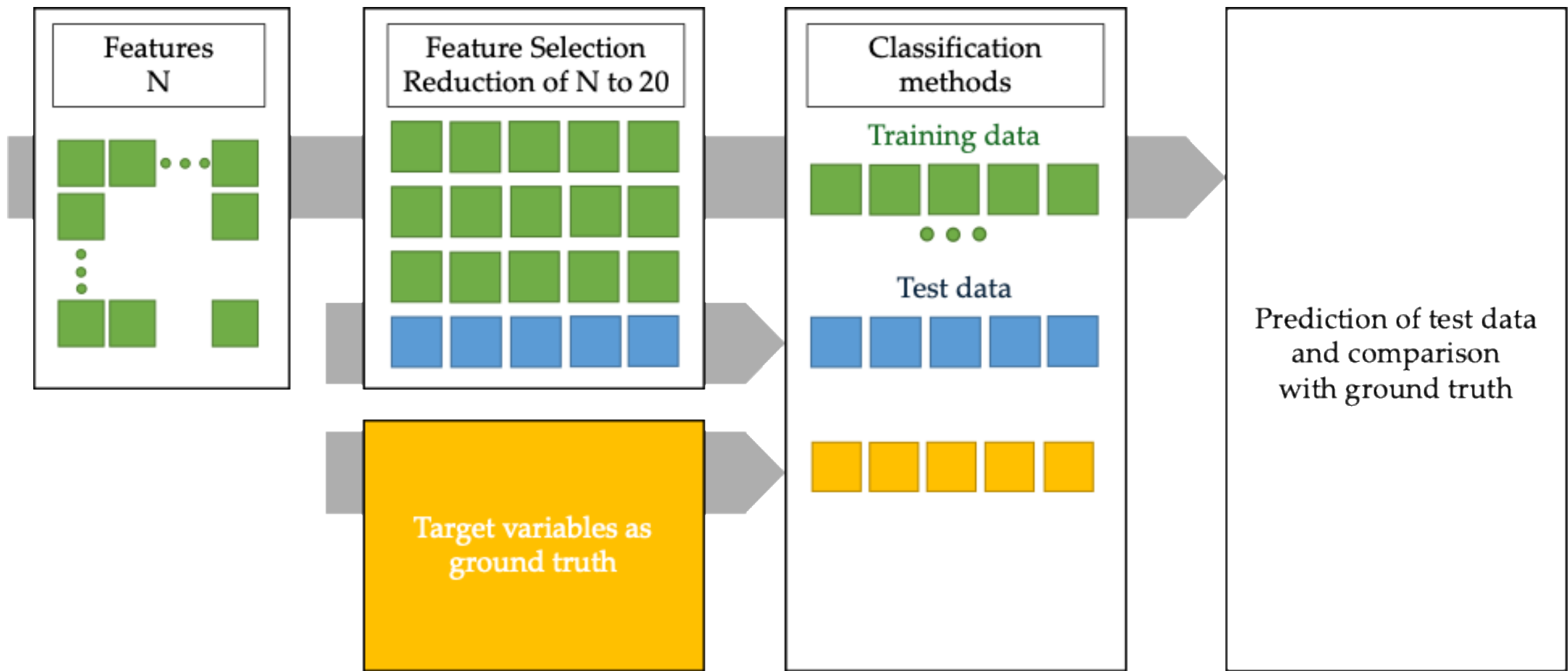

Supplement: Supplementary file 1 [file jcm-15-01359-s001.zip › S1.pdf]

Classifications

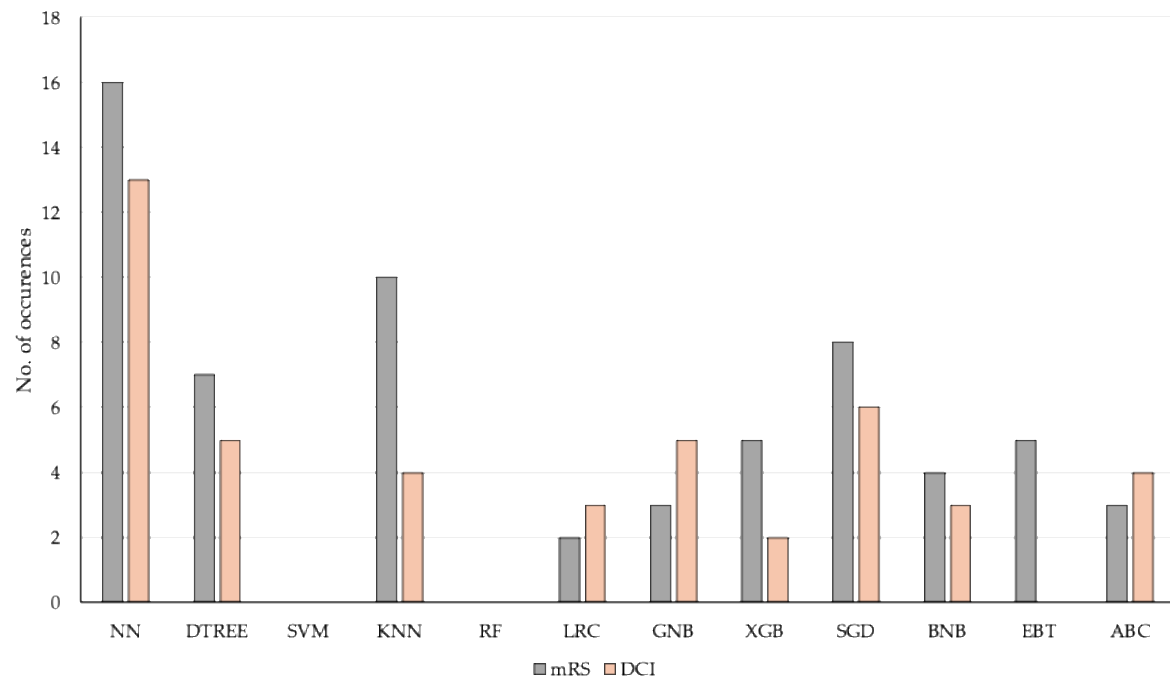

Feature selection

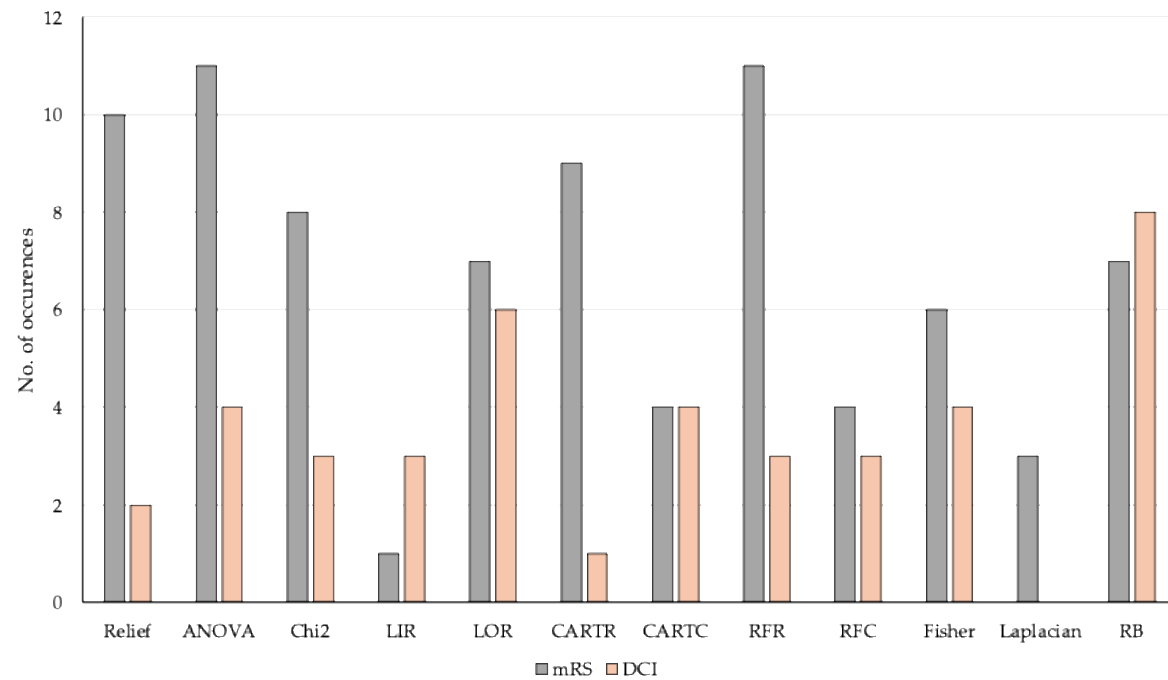

Supplement: Supplementary file 1 [file jcm-15-01359-s001.zip › S2.pdf]
